# Toll-like receptor 3 upregulation in macrophages participates in the initiation and maintenance of pristane-induced arthritis in rats

**DOI:** 10.1186/ar3034

**Published:** 2010-05-25

**Authors:** Liesu Meng, Wenhua Zhu, Congshan Jiang, Xiaojing He, Weikun Hou, Fang Zheng, Rikard Holmdahl, Shemin Lu

**Affiliations:** 1 Department of Genetics and Molecular Biology, Xi'an Jiaotong University School of Medicine, Yanta West Road, Xi'an, Shaanxi 710061, PR China; 2 Key Laboratory of Environment and Genes Related to Diseases (Xi'an Jiaotong University), Ministry of Education, Yanta West Road, Xi'an, Shaanxi 710061, PR China; 3 Division of Medical Inflammation Research, Department of Medical Biochemistry and Biophysics, Karolinska Institute, Alfred Nobels Allé 8, Huddinge, SE-171 77 Stockholm, Sweden

## Abstract

**Introduction:**

Toll-like receptors (TLRs) are involved in both innate and adaptive immune responses and are likely to play a complex role in the pathogenesis of human rheumatoid arthritis (RA) and experimental arthritis. The objective of this study was to identify the key TLR in pristane-induced arthritis (PIA), a rat model for RA, and to clarify its roles in the initiation and maintenance of arthritis.

**Methods:**

Arthritis in DA rats was induced by pristane and the severity was evaluated by macroscopic and microscopic score systems. Spleen TLR and cytokine expression was detected at different time points by real-time polymerase chain reaction (PCR) and flow cytometry. Polyinosine-polycytidylic acid (polyI:C, a ligand of TLR3) or TLR3 specific short-hairpin RNA plasmid for RNA interference was administrated to PIA rats *in vivo*. Serum nitrogen oxide concentration was determined by Griess method, and tumor necrosis factor alpha (TNF-α) was determined by L929 biotest. In splenic macrophages, TLR3 expression was measured by flow cytometry. A rat macrophage cell line (NR8383) was stimulated by pristane, and anti-TLR3 antibody were used to block TLR3 pathway. TLR3 and cytokine expression in NR8383 were detected by real-time PCR.

**Results:**

By screening the TLR expression profile in spleen of DA rats after pristane injection, we found that TLR3 was the most early and prominently upregulated TLR. Both TLR3 mRNA and protein expression of spleen were upregulated at 6 and 26 days after pristane injection. Furthermore, administration of polyI:C exacerbated, whereas RNA interference targeting TLR3 ameliorated, the arthritis. Particularly, TLR3 expression was induced in splenic macrophages of PIA rats, and also in the NR8383 cell line after pristane stimulation in a dose- and time- dependent manner. Upregulation of interferon beta (IFN-β) and TNF-α by pristane stimulation was blocked by anti-TLR3 antibody in NR8383.

**Conclusions:**

TLR3 plays a pivotal role in the initiation and development of PIA which may dependent on macrophage. These findings are useful to understand the pathogenesis of RA and may provide an intriguing therapeutic opportunity for RA.

## Introduction

Rheumatoid arthritis (RA) is an autoimmune chronic inflammatory syndrome affecting 0.5 to 1% of the world population, and is characterized by cellular proliferation in the synovial lining and cartilage and bone destruction of diarthrodial joints [1]. Genetic and serologic evidence in both RA and experimental arthritis favors the involvement of both innate and adaptive autoimmune processes [2,3]. Toll-like receptors (TLRs) are pattern recognition receptors, which form a bridge between innate and adaptive immune systems, and have been considered to be an important factor in the development of RA [4].

TLRs are involved in activation of antigen-presenting cells (APCs) by influencing the uptake and processing of various exogenous and endogenous antigens [5]. Activation of TLRs in APCs not only leads to the upregulation of the costimulatory molecule expression and cytokine secretion [6,7], but also promotes the T cell polarization [8,9]. In addition, TLRs could orchestrate the function of regulatory T cells [10,11].

TLRs are likely to play a complex role in RA, and certain TLRs exhibit a high expression, such as TLR2, 3, 4, 7 in synovium [12-15], TLR3 in fibroblast-like synoviocytes (FLS) [14], TLR2, 4 in CD14^+ ^macrophages and peripheral blood cells from RA [16]. Both exogenous and endogenous TLR ligands have been detected in synovia, synovial fluids and sera of RA patients [14,17,18]. Importantly, these ligands are capable of stimulating FLS and/or immunocytes via triggering TLRs to produce proinflammatory cytokines [12,19-24], and may also activate the autoreactive T and B cells [25-27]. In particular, injection of TLR2 and TLR9 ligands, peptidoglycan (PGN) and CpG DNA, into articular cavities induces arthritis in mice [28,29].

Based on the above-mentioned reasons, it seems that TLRs play essential roles in the pathogenesis of RA. However, most studies on RA are descriptive and focus on TLR2 and 4 in synovium. Thus, a systemic study about TLR roles in the initiation of immune response of RA will provide new insight for elucidating the pathogenesis of RA. The TLR genes are highly conserved and using animal models should be reasonable to address the issue of TLR roles in RA development.

Various animal models represent different or partly different disease processes of RA, and a few studies have concerned TLR roles in the pathogenesis of arthritis. TLR2 and 4 were reported to be involved in the chronicity and erosive destruction of streptococcal cell wall induced arthritis and spontaneous arthritis in IL-1 receptor antagonist-knockout mice [8,30,31]. TLR4 takes part in the initiation and progression of both autoantibody/lipopolysaccharide (LPS)-triggered arthritis and serum transferred arthritis [32,33]. It has also been suggested that cathepsin K could ameliorate mycobacteria induced arthritis in rats through suppression of TLR9 signaling in dendritic cells (DCs) [34]. Of particular interest is the pristane-induced arthritis (PIA) model as it is induced with a single subcutaneous injection with a well-defined compound lacking antigenic properties. The induced disease is joint specific and fulfills the clinical criteria of RA [35,36]. Importantly, the arthritogenic adjuvant stimuli may mimic such a trigger in humans represented by an infectious agent or a non-specific inflammatory response. It is known that pristane induces a strong innate immunity through the triggering of IL-6 production [37], suggesting that TLRs may participate in and mediate the immune response. Altogether, the mechanism of TLRs involved in experimental arthritis is not understood completely.

In the present study, we tried to find the key TLR in arthritis development by screening the TLR1-9 expression profile in spleen from PIA rats. The results indicated TLR3 showed a unique expression pattern compared with other TLRs in spleen of PIA rats. And in PIA rats treated with polyinosine-polycytidylic acid (polyI:C) or RNA interference (RNAi) of TLR3, arthritis manifestations could be modified. Furthermore, TLR3 may exert its influence in a macrophage-dependent manner. These data illustrated the crucial role of TLR3 in the initiation and development of PIA.

## Materials and methods

### Animals

DA rats were bred in a specific pathogen-free animal house. Each group contained 8 to 10 rats at the age of 8 to 12 weeks. The experiments were approved by the Institutional Animal Ethics Committee of the University (permission No. 2006-21).

### Induction and evaluation of arthritis

Arthritis was induced by a single subcutaneous injection with 300 μl of pristane (ACROS Organics, Morris Plains, NJ, USA) at the base of the rat's tail. Arthritis development and severity were monitored every two to four days by the perimeters of ankle and mid-paw, and a macroscopic scoring system as described [35].

### Histopathological examination of joints

Left ankle joints of rats were sectioned and stained with hematoxylin and eosin. Pathological severities were estimated by extent of 10 items: a. thickness of synovium lining layer; b. pannus; c. synovium inflammatory cells; d. angiogenesis; e. cartilage erosion; f. bone erosion; g. joint ankylosis; h. change of overall articular structure; i. new bone formation; and j. new cartilage formation. Each pathological item was scored on a scale consisting of score 0 (normal) to 3 (most severe). The maximum histopathological scores of arthritis are 30 for each ankle. Synovitis was estimated by addition of item a. to d. scores; joint destruction by e. to h. scores; joint healing by i. and j. scores.

### RNA quantitation

Total RNA was isolated with TRIzol^® ^Reagent (Invitrogen, Carlsbad, CA, USA), and cDNA was synthesized by First Strand cDNA Synthesis Kit (Fermentas, Burlington, ON, Canada). Real-time quantitative PCR was performed by iQ5 (BIO-RAD, Hercules, CA, USA) with SYBR^® ^Premix *Ex Taq*™ II (TaKaRa, Ohtsu, Shiga, Japan) for TLR and cytokine mRNA quantitation. And gene expression was normalized by β-actin. The information on primers, products and annealing temperatures were depicted in Table 1.

**Table 1:** Information of primers for conventional PCR and Real-time PCR

Gene name	NCBI Accession No.	Sequence(5'-3')		Size (bp)	annealing temperature (°C)
*TLR1*	NM_001172120	forward	CAGCAGCCTCAAGCATGTCTA		
		reverse	CAGCCCTAAGACAACAATACAATAGAAGA	82	60
*TLR2*	NM_198769	forward	CTCCTGTGAACTCCTGTCCTT		
		reverse	AGCTGTCTGGCCAGTCAAC	74	60
*TLR3*	NM_198791	forward	GATTGGCAAGTTATTCGTC		
		reverse	GCGGAGGCTGTTGTAGG	205	54
*TLR4*	NM_019178	forward	GATTGCTCAGACATGGCAGTTTC		
		reverse	CACTCGAGGTAGGTGTTTCTGCTAA	135	54
*TLR5*	XM_001063885	forward	GGGCAGCAGAAAGACGGTAT		
		reverse	CAGGCACCAGCCATCCTTAA	61	60
*TLR6*	NM_207604	forward	AGAACCTTACTCATGTCCCAAAAGAC		
		reverse	AGATCAGATATGGAGTTTTGAGACAGACT	79	60
*TLR7*	NM_001097582	forward	GTTTTACGTCTACACAGTAACTCTCTTCA		
		reverse	TTCCTGGAGGTTGCTCATGTTTT	75	60
*TLR8*	NM_001101009	forward	GGGGTAACACACCGTCTA		
		reverse	GTCAAGGCGATTTCCACT	150	60
*TLR9*	NM_198131	forward	CCGAAGACCTAGCCAACCT		
		reverse	TGATCACAGCGACGGCAATT	70	60
*TNF-α*	NM_012675	forward	TCAGCCTCTTCTCATTCCTGC		
		reverse	TTGGTGGTTTGCTACGACGTG	203	60
*IL-6*	NM_012589	forward	AAGAAAGACAAAGCCAGAGTC		
		reverse	CACAAACTGATATGCTTAGGC	263	60
*IFN-γ*	NM_138880	forward	CCCTCTCTGGCTGTTACTGC		
		reverse	TTTCGTGTTACCGTCCTTTTG	149	54
*IFN-β*	NM_019127	forward	CTTGGGTGACATCCACGACTAC		
		reverse	GGCATAGCTGTTGTACTTCTTGTCTT	92	54
*β-actin*	NM_031144	forward	GAGGGAAATCGTGCGTGAC		
		reverse	GCATCGGAACCGCTCATT	157	60
*gfp*	-	forward	GGCACCGATTTCAAGGAG		
		reverse	CGCCGATGGGGGTATTCT	190	54

### Fluorescence-activated cell sorting (FACS) analysis of TLR expression in splenocytes

Three tubes containing 10^6 ^splenocytes for cell surface TLR detection were incubated with TLR2, TLR3 and TLR4 antibodies (Santa Cruz Biotechnology, Santa Cruz, CA, USA) respectively. In the other tube for intracellular TLR3, the cells were fixed and perforated before TLR3 antibody incubation. For detection of macrophage TLR3, two tubes containing 10^6 ^splenocytes were incubated with HIS36-PE (anti-rat macrophage, eBioscience, San Diego, CA, USA) firstly. And for cell surface TLR3 detection, the cells were incubated with TLR3 antibody at the same time. For intracellular TLR3 detection, the cells in the other tube after His36-PE staining, were fixed and perforated before TLR3 antibody incubation. The cells from both tubes were incubated with goat anti-rabbit IgG-biotin followed by streptavidin-cychrome. Cells were analyzed by using a FACScanto (Becton Dickinson, Franklin Lakes, NJ, USA), and datum analysis was performed using FlowJo software (Tree Star, Ashland, OR, USA).

### Administration of TLR ligands to PIA rats

Thirty-six rats were randomly divided into four groups. The control group was injected subcutaneously with 300 μl phosphate buffered saline (PBS) only. Other groups were injected subcutaneously with 300 μl pristane, then administered subcutaneously with 150 μl saline containing 100 μg polyI:C (Amersham Biosciences, Piscataway, NJ, USA) in the polyI:C group, 50 μg LPS (Sigma-Aldrich, St. Louis, MO, USA) in the LPS group, and none in the saline group, respectively, at two days after pristane injection [38,39].

### Cell transfection and treament with *TLR3 *RNAi *in vivo*

The target sequences of *TLR3 *gene are GAA TCA CAT CTC GAA GAT A, called as shRNA1-TLR3; CTC TCA ATT TAA CGA AGA A, as shRNA2-TLR3; ACC TCG ACC TCA CAG AGA A, as shRNA3-TLR3; and CCT GAC AGA GCT CCA TCT A as shRNA4-TLR3. The sequence of negative control shRNA is TTC TCC GAA CGT GTC ACG T. These different short-hairpin RNA (shRNA) sequences were inserted into pGCsilencer™ U6/Neo/GFP/RNAi plasmid (Genechem, Shanghai, China). The plasmids were extracted with E.Z.N.A.™ Endo-free Plasmid Maxi Kit (Omega Bio-tek, Doraville, GA, USA), and NR8383 cells were transfected with Lipofectamin™ 2000 (Invitrogen). TLR3 expression of cells was detected by RT-PCR after 24 hours and by FACS after 48 hours. DA rats randomly divided into three groups were injected subcutaneously with 300 μl pristane. At one day before pristane injection, and 5, 11 and 17 days after pristane injection, 1 ml of saline was injected peritoneally into the rats in the saline group; 50 μg shRNA3-TLR3 in 1 ml of saline in the shRNA-TLR3 group; and 50 μg negative control shRNA in 1 ml of saline in the shRNA-NC group [40,41]. At 20 days after pristane injection (d20), GFP mRNA expression was detected by RT-PCR to monitor the plasmid presence in spleen (Primer information was depicted in Table 1). And TLR3 mRNA expression in the spleen was detected by real-time quantitative PCR for determination of the RNAi effectiveness.

### Measurement of NO concentration

Serum protein was removed, and NO_3_^- ^was reduced to NO_2_^- ^by using cadmium filings. Then NO_2_^- ^was measured by a microplate assay method based on the Griess reaction.

### Measurement of TNF-α concentration

The mouse fibroblast cell line, L929, was cultured in RPMI 1640 (Hyclone, Logan, UT, USA) containing 10% FCS. Ten thousand cells in 100 μl were incubated for 24 hours. Then sera containing 1 mg/ml actinomycin D were added to replace the original medium. After an 18-hour incubation, cell viability was evaluated by the methyl thiazolyl tetrazolium method. Rat recombinant TNF-α (Bender MedSystems Inc., Burlingame, CA, USA) was used as the standard.

### Stimulation of macrophages with pristane

The rat macrophage cell line, NR8383, was cultured in F-12 K medium (Sigma-Aldrich) with 20% FBS (Hyclone). Emulsion of pristane was made by repeated aspiration with PBS. Half-million cells per well were put in a six-well plate, then a 50 μl pristane emulsion was added in the culture medium. The theoretical pristane concentration is 0.1, 1, 10, 100 μM, 1 mM and 10 mM, calculated on the basis of original volume of pristane employed. The cells were collected after 8, 24, 48 and 72 hours of stimulation.

### TLR3 blockade assay in NR8383

NR8383 were seeded in six-well plates at a density of half-million per well. In the TLR3 Blockade assay, cells were treated with anti-TLR3 or isotype control antibodies at a concentration of 20 μg/ml for 90 minutes before being stimulated with 100 μM pristane or 10 μg/ml polyI:C. The cells were collected after 24 hours' stimulation.

### Statistics

Quantitative data were expressed as means ± SEM. The statistical analysis of differences between experimental groups was performed by Mann-Whitney U test or Student's *T *test. Correlation analysis was performed by Pearson test. *P *< 0.05 was considered significant.

## Results

### TLR3 expression of spleen exhibits a remarkable increase in the PIA rat model

**Figure 1:**
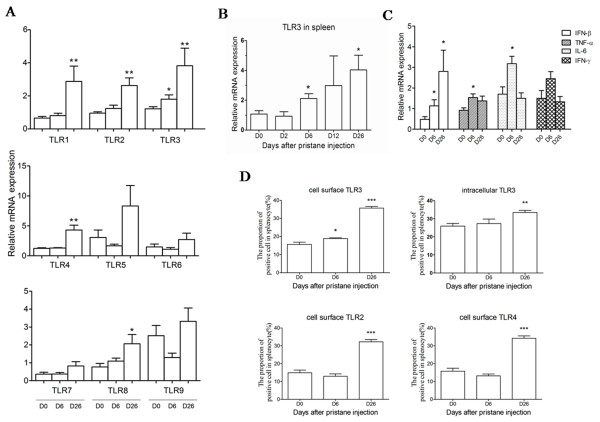
**TLR and cytokine expression profile of spleen from DA rats with pristane-induced arthritis**. The mRNA expression **(a) **of TLR1-9 at d0, 6 and 26, **(b) **of TLR3 at d0, 2, 6, 12 and 26, **(c) **and of cytokines at d0, 6 and 26 in spleen of rats was measured by Real-time quantitative PCR (n = 8 to 10 for each time point). Relative mRNA expression was compared with β-actin. Protein expression of TLRs in spleen was detected by FACS at d0, 6 and 26 (n = 3 for each time point). The positive splenocyte proportion **(d) **of cell surface and intracellular TLR3, and cell surface TLR2 and TLR4 is showed in the course of arthritis. Data were collected after correction for isotype-matched IgG control. Values are shown as means ± SEM. Levels of significance between the pristane treated group (6, 26 days) and untreated group (0 day) were calculated by using Mann-Whitney U test (* *P *< 0.05, ** *P *< 0.01, *** *P *< 0.001).

Arthritis-susceptible DA rats were given a single subcutaneous injection of pristane known to induce arthritis within two weeks [35]. The spleens were isolated and analyzed for mRNA expression at different time points. The results showed that only TLR3 mRNA expression was upregulated as early as six days after pristane injection (d6) (Figure 1a). TLR1-4 and TLR8 were significantly expressed at 26 days after pristane injection (d26) (Figure 1a). However, TLR5, 6, 7, 9 did not show a significant change after pristane injection. Another independent experiment was performed with more time points, 2 and 12 (d12) days after pristane injection. TLR3 expression increased dynamically and progressively with the arthritis initiation and development. It was significantly upregulated at d6 and d26, and had a high level at d12 (Figure 1b). IFN-β, known to be triggered by TLR3, was upregulated at d6 and d26 (Figure 1c). Of the proinflammatory cytokines, TNF-α and IL-6 mRNA expression only increased at d6 (Figure 1c). And IFN-γ mRNA expression at d6 and d26 had no difference comparing with control group (d0).

Based on the expression at mRNA level, we determined the protein expression of TLR2, 3 and 4 in the spleens of PIA rats by flow cytometric staining. The results showed that the proportion of cell surface TLR3 positive cells increased significantly at d6 and d26, while that of intracellular TLR3 positive cells and cell surface TLR2 and 4 positive cells increased significantly only at d26 (Figure 1d).

### The administration of TLR3 ligand increases arthritis severity

**Figure 2:**
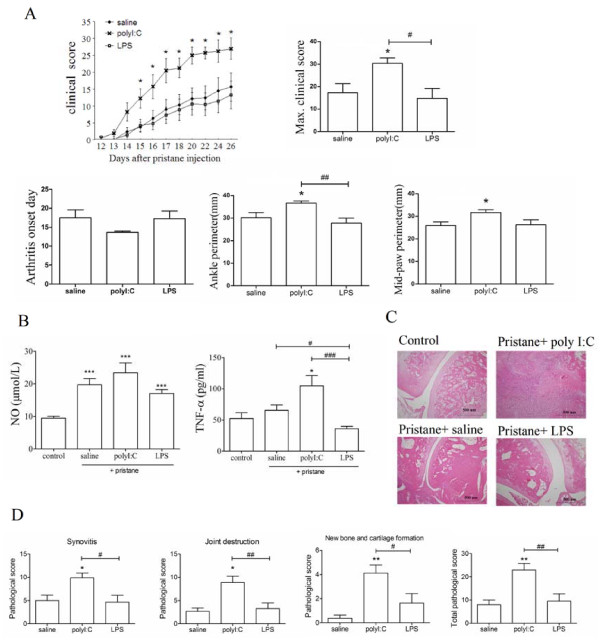
**More severity, higher serology index and increased histopathological scores of PIA caused by polyI:C treatment**. The arthritis clinical indexes **(a) **including clinical score, max clinical score, onset day, mid-paw perimeter, ankle perimeter; and serum NO and TNF-α concentration at d26 **(b) **were compared among the polyI:C, LPS and saline groups. **(c)**, In representative histological images of ankle joints, the ankle joints in the control group and PIA groups treated with saline or ligands were stained by H&E. **(d)**, For the histological analysis of synovitis, joint destruction, new bone and cartilage formation and the total histological index, the various scores were quantified among PIA groups treated with saline and ligands. Data are presented as means ± SEM. * represents the comparison between ligands treated groups and saline group in A and D, or between ligands treated groups and control group in B. But ^# ^between ligands treated groups as marked. Levels of significance were calculated using Mann-Whitney U test (n = 9 for each group, */^# ^*P *< 0.05, **/^## ^*P *< 0.01, ***/^### ^*P *< 0.001).

The pristane pre-injected rats were treated with ligands of TLR3 (polyI:C), TLR4 (LPS) and saline, respectively. The clinical score of the polyI:C group showed significant increase compared with the saline group and the LPS group from 15 days after pristane injection, and maximum clinical score of the polyI:C group consistently had a significant difference compared with other groups (Figure 2a). Maximum perimeters of hind paw and ankle also increased in the polyI:C group (Figure 2a). Serum NO concentration increased significantly in all PIA groups, in particular in the polyI:C group (Figure 2b), but there are no differences among all pristine-treated groups. TNF-α concentration in the polyI:C group increased significantly compared with other groups (Figure 2b).

Histopathologically, polyI:C administration aggravated the pathologic changes with abundant inflammatory cell infiltrates and bone destruction (Figure 2c). PolyI:C group showed significant differences with the LPS group and the saline group in the pathological score of synovitis, joint destruction, new bone and cartilage formation (Figure 2d). Meanwhile, the total pathological score of polyI:C group had significantly increased compared with other groups (Figure 2d). All results from the clinical, serological and pathological data indicated that TLR3 ligand administration to the PIA rats could make the disease more severe, and further highlighted the important role of TLR3 in PIA development. Then RNAi of TLR3 *in vivo *was performed to convince the authors that TLR3 is essential in PIA pathogenesis.

### The RNAi of TLR3 significantly ameliorates PIA in rats

**Figure 3:**
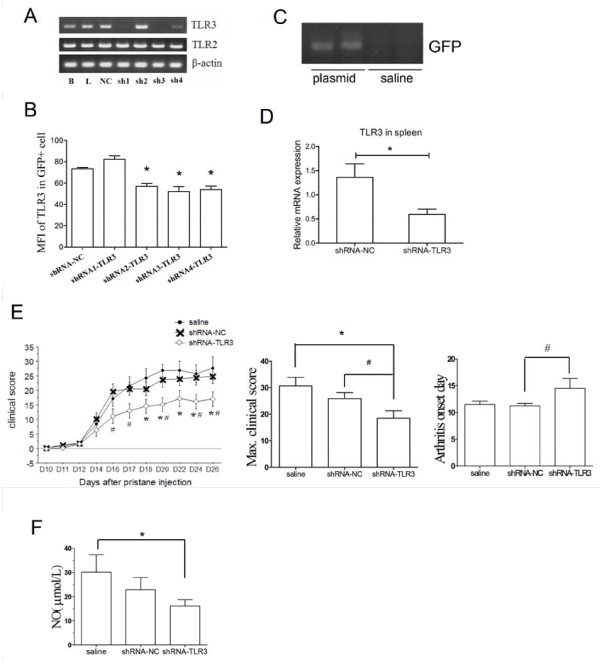
**Attenuated severity and reduced and a serum NO concentration of PIA caused by RNAi of TLR3 *in vivo***. Four TLR3-shRNA plasmids and a negative control plasmid were transfected into NR8383 cells. mRNA (**(a)**, 24 hours after transfection) and protein (**(b)**, 48 hours after transfection) expression levels of TLR3 were detected by RT-PCR and FACS respectively, and shRNA3-TLR3 (shRNA-TLR3) shows the best RNAi efficacy. The plasmids of shRNA-TLR3 and shRNA-NC were used to treat PIA rats, and GFP mRNA expression **(c) **in plasmid treatment groups except in the saline group was successfully amplified by RT-PCR. **(d)**, TLR3 expression of the spleen at d20 was detected by Real-time quantitative PCR. Relative mRNA expression was compared with the housekeeping gene β-actin. The arthritis clinical indexes **(e) **including clinical score, max clinical score and onset day; and serum NO concentration at d26 **(f) **were compared among three group PIA rats treated with saline, shRNA-NC and shRNA-TLR3, respectively. Data are presented as means ± SEM. * represents the comparison between the shRNA treated group and the saline group, and ^# ^between the shRNA-TLR3 and shRNA-NC groups. Levels of significance were calculated by using Mann-Whitney U test (n = 8 for each group, *^/# ^*P *< 0.05).

NR8383 macrophages were transfected with four different TLR3 shRNA plasmids to determine whether the expression of TLR3 could be downregulated. Results from RT-PCR and flow cytometric staining showed that shRNA3-TLR3 most efficiently downregulated TLR3 mRNA and protein expression (Figure 3a, b). The plasmids, shRNA3-TLR3 (shRNA-TLR3 group) and shRNA-NC (shRNA-NC group, as a negative control), were applied to PIA rats *in vivo*. The GFP gene in the plasmid treatment groups, except for the saline group, was successfully amplified by RT-PCR (Figure 3c), which showed that plasmids with peritoneal injection in rats could accumulate in the spleen. And TLR3 mRNA expression in the spleen of the shRNA-TLR3 group was significantly lower than that of the shRNA-NC group at d20 (Figure 3d). The clinical score of the shRNA-TLR3 treated rats was lower than that of the shRNA-NC and saline treated rats from 16 days after pristane injection (Figure 3e). The PIA onset day of the shRNA-TLR3 group was later than that of the other two groups (Figure 3e). The shRNA-TLR3 group showed a significantly lower maximum score compared with the other two groups (Figure 3e). The NO concentration in the shRNA-TLR3 group was significantly lower than that of the saline group (Figure 3f). Results indicated that the knockdown of TLR3 expression can ameliorate the disease after pristane injection in rats.

### TLR3 expression was induced in macrophages with pristane stimulation

**Figure 4:**
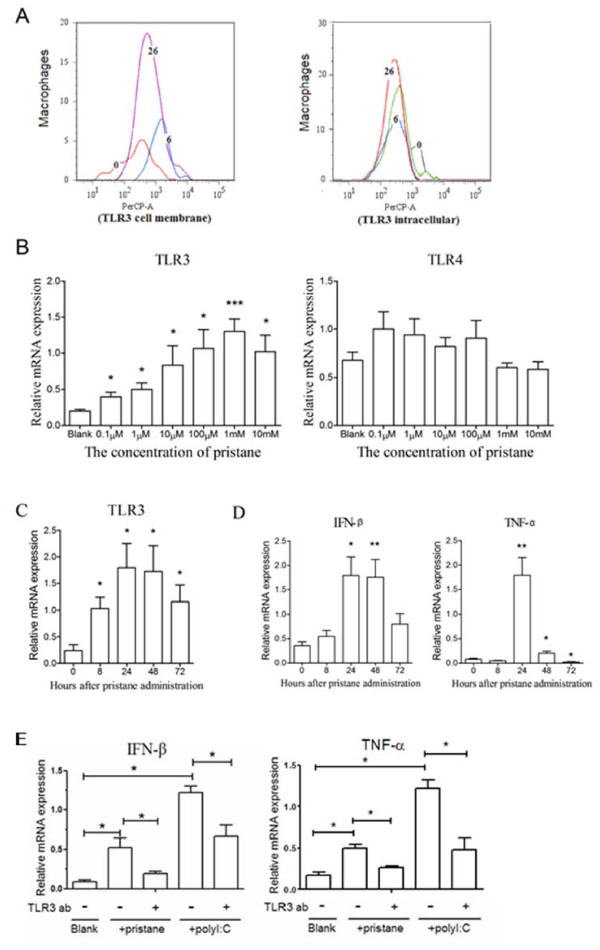
**TLR3 expression was induced in macrophages with pristane stimulation**. In splenic macrophages, TLR3 protein expression was detected by FACS at 0, 6 and 26 days after pristane injection. The proportion and mean fluorescence intensity (MFI) of cell surface and intracellular TLR3 were detected on His36^+ ^(anti-rat macrophage) gated TLR3^+ ^cells from splenocytes, and representative histograms **(a) **are showed in the course of arthritis. Data were collected after correction for isotype-matched IgG control. NR8383 cells were stimulated with a series of concentrations of pristane (0.1 μ, 1 μ, 10 μ, 100 μ, 1 m, 10 mM as theoretical pristane concentration), and TLR3 and TLR4 mRNA expression was detected at 48 hours after stimulation **(b)**. The expression of TLR3 increased with a raised dosage of pristane (r^2 ^= 0.78) by Pearson correlation analysis. With 100 μM pristane stimulation, TLR3 **(c) **and IFN-β, TNF-α **(d) **mRNA expression was measured at 0, 8, 24, 48 and 72 hours. **(e) **NR8383 pretreated with anti-TLR3 or isotype control antibodies were stimulated by 100 μM pristane or 10 μg/ml polyI:C, and IFN-β, TNF-α mRNA expression was measured at 24 hours. mRNA expression level was detected by Real-time quantitative PCR, and data are presented as means ± SEM of four replicated determinations from three independent experiments. * represents the comparison with the control group (Blank in *B *and *E*, and 0 hour in *C *and *D*), and levels of significance were calculated by using Student's *T *test (* *P *< 0.05, ** *P *< 0.01, *** *P *< 0.001).

TLR3 expression in splenic macrophages was detected by flow cytometric staining in PIA rats. The mean fluorescence intensity (MFI) of cell surface TLR3 positive cell increased markedly at d6 and the proportion increased significantly at d26, although the intracellular TLR3 expression showed no change after pristane injection, suggesting that the role of TLR3 in the pathogenesis of the disease seems to be associated with the high expression on the macrophage membrane (Figure 4a).

To confirm the role of pristane on TLR3 expression upregulation in macrophages, we stimulated NR8383, a rat macrophage cell line, with pristane. We found that TLR3 mRNA expression was increased in a dose-dependent manner after pristane stimulation, and reached the peak at 1 mM pristane (Figure 4b). In contrast, TLR4 mRNA expression did not show a dose-dependent tendency. TLR3 expression was induced already at 8 hours after pristane stimulation, and was highest at 24 hours (Figure 4c). IFN-β mRNA expression also showed a pattern similar to TLR3, and increased significantly in the group stimulated at 24 and 48 hours (Figure 4d). TNF-α mRNA expression increased significantly at 24 and 48 hours (Figure 4d). However, as NR8383 were pretreated with anti-TLR3 antibody, the upregulation of IFN-β and TNF-α expression stimulated by both pristane and polyI:C was inhibited significantly (Figure 4e). The result indicated that in macrophages pristane certainly orchestrated high TLR3 mRNA expression, which activated the downstream cytokine pathway.

## Discussion

A single subcutaneous injection of pristane in DA rats leads to chronic relapsing arthritis similar to RA in humans. We now show that pristane injection also leads to an upregulated expression of TLRs, in particular TLR3. TLR3 was found to play a critical role in the development of arthritis as an injection of TLR3 ligand enhanced arthritis and downregulation of TLR3 through treatment with RNAi alleviated arthritis. And TLR3 expression was induced with pristane stimulation particularly in macrophages.

PIA is a T cell dependent disease, which can be transferred with αβT cells [42], and there is no evidence so far arguing for a pathogenic role of B cells. In the human disease it is clear at present that many patients benefit from depletion of B cells through injection of antibodies to CD20. In contrast, in PIA antibodies to citrullinated proteins are not evoked and there is no evidence for pathogenic antibodies mediating the disease, unlike in the collagen-induced arthritis model. However, the immune response in PIA also shows some striking similarities with RA since rheumatoid factors and antibodies to RA33 are produced in both diseases [43]. Importantly, a strong activation of an innate response is apparent in both RA and PIA, although in RA it has been possible to analyze the response only in already established disease. In RA synovial tissues a strong overexpression of TLR3 and 4 has been reported, whereas isolated macrophages show high expression of TLR2 and 4 [13,16]. But there has been no study on TLRs in the PIA rat model so far.

A subcutaneous injection of mineral oil or phytol, structurally similar to pristane, leads to an accumulation of oil at the injection site but also a rapid spread of small volumes to draining lymph nodes and subsequently spreads systemically, reaching the spleen [44,45]. Previous studies have shown that T cells isolated from spleens, less than eight days after pristane injection, transfer severe arthritis, showing that the critical arthritogenic change induced by pristane is present in the spleen [42]. Therefore we selected the spleen to study the innate immune reaction after pristane injection.

Screening TLR mRNA expression at different time points after pristane injection, we found that expression of TLR1, 2, 3, 4 and 8 in the spleen is upregulated in the development stage of arthritis (d26), which is consistent with previous studies from arthritis models and RA patients [20,22,23,46]. A compelling TLR is TLR3 as it was highly expressed uniquely in the early phase, and increased progressively with the development of the disease. It has been reported that TLR3 is overexpressed in synovial tissue from both early and longstanding RA [12,13]. A possible pathogenic role of the upregulated TLR3 could be that it recognizes RNA released from necrotic synovial fluid cells, and then activates RA synovial fibroblast [14]. TLR3 combined with TLR4, TLR7/8 is also involved in the regulation of DC activation and cytokine production in RA patients [12]. It indicates that TLR3 in the spleen may play a vital role in PIA. To examine whether the observed TLR overexpression has functional consequences, downstream cytokines of TLR pathway, such as TNF-α, IL-6, IFN-γ and IFN-β mRNA expression in the spleen were determined. IFN-β expression was found to increase at both d6 and d26, whereas TNF-α and IL-6 expression only increased at d6, and the IFN-γ expression level did not change. These proinflammatory cytokines are known to be regulated by many TLRs via several intracellular pathways but are mainly associated with NF-κB. TLR3 and possibly TLR4 activate interferon regulatory factor 3 which regulates IFN-β expression [47]. By flow cytometric staining we confirmed high TLR3 protein expression in splenocytes. The expression of TLR3 on splenocyte surfaces increased at d6 and d26, reflecting its mRNA expression pattern.

Based on the experimental data from the gene expression profile and TLR2, 3, 4 protein expression during the disease course, we suggest that TLR3 plays an important role in the pathogenesis of PIA. Such a role could be confirmed through *in vivo *experiments with TLR3 ligands and RNAi. In a previous study, intra-articular treatment with polyI:C to rats caused a rapid and strong synovitis [48], which suggested the important role of TLR3 signaling in arthritis pathogenesis. And in our research, the systemic treatment with polyI:C synergism with pristane showed that TLR3 ligand could exacerbate arthritis. This dominant role of the TLR3 ligand was also proved by pathology of the PIA rat ankles. The possible mechanism of polyI:C's effect on PIA could be that more TLR3 is activated by polyI:C, which triggers more severe inflammatory reaction in joints. Meanwhile, the results also emphasize the important roles of the ligand in arthritis pathogenesis. But at present, it is still not defined which endogenous ligand of TLR3 participates in PIA. Thus, priority should be given to exploring the probable ligand and investigating its roles in arthritis.

A crucial role of TLR3 in the PIA pathogenesis could also be confirmed by treatment with RNAi *in vivo*. The shRNA plasmids did not show any immunostimulatory effect *in vivo *because the shRNA-NC group shows no differences from the saline group in all indexes. The results indicated that the RNAi of TLR3 participated in the initiation of PIA significantly delayed the onset day and decreased arthritis severity. NO concentration of shRNA-TLR3 group was also significantly lower than that of the shRNA-NC and saline groups. The result validates the importance of TLR3 in PIA, however, the ligands and the functional consequences of TLR activation in PIA are not yet fully understood.

TLR3 expresses mainly on DC for humans but on macrophages for mice [49]. It has been found that macrophages are involved in the pathology of K/BxN serum-induced arthritis [50]. Macrophage-derived reactive oxygen species (ROS) participate in T cell selection, maturation and differentiation, and also mediate protection against arthritis by suppressing T cell activation [51]. In addition, macrophages stimulated by excessive interleukin-15 may activate the characteristic autoreactive CD4^+ ^T cells in RA [27]. The increased macrophage activation may be mediated by TLRs in RA or other autoimmune inflammatory diseases [16,52]. All findings indicate macrophages are a main player in the development of arthritis. We found that TLR3 positive macrophages were increased in the spleen of PIA rats, and cell surface TLR3 showed high expression in the initiation stage of arthritis. It suggests that cell surface TLR3 on macrophage, but not the intracellular expression, exerts a crucial influence on the pathogenesis of PIA.

Using a rat macrophage cell line, NR8383, we could confirm that TLR3, but not TLR4, was upregulated after stimulation with pristane, which excludes LPS contamination in the experiment. Furthermore, the mRNA expression of IFN-β, the main downstream cytokine of TLR3, also increased with the same tendency as that of TLR3. The increase of IFN-β and TNF-α was inhibited by anti-TLR3 antibody, suggesting that this induction was TLR3 dependent. The above mentioned data indicate TLR3 and its downstream molecules in macrophage were activated by pristane, and then took part in the pathogenesis of PIA. More TLR3 expression on macrophages could affect the cell function, such as the capacity of antigen presentation, ROS production, phagocytosis, secretion of cytokines and chemokines and so on. Macrophages with upregulated TLR3 may affect the balance of Th1/Th2 and the activations of autoreactive T cells and B cells [25,42].

However, it is still unclear how pristane induces TLR3 expression. The interaction between TLR and alkanes like pristane is not completely known. It is reported that oxidized alkane polymers induced activation of the TLR1/2 pathway as its ligands. And pristane could augment the effect of TLR7 ligands but does not directly activate TLR7. In a PIA rat model, further study is needed as to whether pristane or its metabolism products are the ligand of TLR3, or if it induces TLR3 by another pathway. Further studies are also needed to understand underlying mechanisms as to how TLR3 activates joint-specific autoimmune T cells via macrophages.

## Conclusions

In summary, we screened TLR and downstream cytokine expression in the spleen of PIA rats, and found that only TLR3 expression was increased at the initiation stage. Regulating TLR3 by using ligand or shRNA *in vivo *could directly aggravate or relieve symptoms of arthritis. TLR3 may play its role via splenic macrophage, since it had a high expression on macrophages after pristane injection *in vivo*. And the induction of TLR3 was specifically due to pristane stimulation in macrophage cell lines with a dose- and time- dependent manner. These findings are not only useful in understanding the underlying pathogenesis of RA, but also provide a valuable clue for other immunological disease research.

## Abbreviations

APC: antigen-presenting cell; DC: dendritic cell; FACS: fluorescence-activated cell sorting; FLS: fibroblast like synoviocyte; IFN-β: interferon beta; LPS: lipopolysaccharide; MFI: mean fluorescence intensity; PBS: phosphate buffered saline; PGN: peptidoglycan; PIA: pristane-induced arthritis; polyI:C: polyinosine-polycytidylic acid; RA: rheumatoid arthritis; RNAi: RNA interference; ROS: reactive oxygen species; shRNA: short-hairpin RNA; TLR: toll-like receptor; TNF-α: tumor necrosis factor alpha.

## Competing interests

The authors declare that they have no competing interests.

## Authors' contributions

LM, WZ, SL and RH conceived and designed the experiments. LM and WZ performed the experiments and analyzed the data. CJ and XH performed some experiments. WH and FZ prepared reagents. LM, WZ, RH and SL had extensive scientific discussion for this study and wrote the paper. All authors read and approved the final manuscript.
